# 8-{1-[(4′-Fluoro-[1,1′-biphen­yl]-4-yl)meth­yl]piperidin-4-yl}-3,4-di­hydro­quinolin-2(1*H*)-one chloro­form 0.25-solvate

**DOI:** 10.1107/S160053681303448X

**Published:** 2014-01-04

**Authors:** Nisar Ullah, Helen Stoeckli-Evans

**Affiliations:** a Department of Chemistry, King Fahad University of Petroleum & Minerals, 31261 Dahran, Saudi Arabia; b Institute of Physics, University of Neuchâtel, rue Emile-Argand 11, CH-2000 Neuchâtel, Switzerland

## Abstract

In the asymmetric unit of the title compound, C_27_H_27_FN_2_O·0.25CHCl_3_, there are two independent mol­ecules (*A* and *B*) together with a partially disordered chloro­form mol­ecule situated about an inversion center. The conformation of the two mol­ecules is very similar. The bridging piperidine rings each have a chair conformation while the piperidin-2-one rings of the quinoline moiety have screw-boat conformations. The benzene rings of the biphenyl moiety are inclined to one another by 26.37 (4) and 23.75 (15)° in mol­ecules *A* and *B*, respectively. The mean plane of the central piperidine ring [r.m.s. deviation = 0.241 (2) Å in both mol­ecules *A* and *B*] is inclined to the benzene ring of the quinoline moiety by 80.06 (4) in *A* and 83.75 (15)° in *B*, while it is inclined to the adjacent benzene ring of the biphenyl group by 73.623 (15) in *A* and 75.65 (14)° in *B*. In the crystal, individual mol­ecules are linked by pairs of N—H⋯O hydrogen bonds, forming *A*–*A* and *B*–*B* inversion dimers with *R*_2_^2^(8) ring motifs. The dimers are stabilized by C—H⋯O hydrogen bonds and linked *via* C—H⋯F and C—H⋯N hydrogen bonds into a three-dimensional network. Several C—H⋯π inter­actions are also present.

## Related literature   

For the synthesis and dual D_2_ and 5-HT_1A_ receptor binding affinities of 5-piperidinyl and 5-piperazinyl-1*H*-benzo[*d*]imid­azol-2(3*H*)-ones, see: Ullah (2013 ▶). For the synthesis of new 4-aryl-1-(bi­aryl­methyl­ene)piperidines, structural analogs of Adoprazine (SLV313), see: Ullah & Al-Shaheri (2012 ▶). For the synthesis of the title compound, see: Ullah (2012 ▶) and Eastwood (2000 ▶). For standard bond-length data, see: Allen *et al.* (1987 ▶). For a description of hydrogen-bond motifs, see: Bernstein *et al.* (1995 ▶)
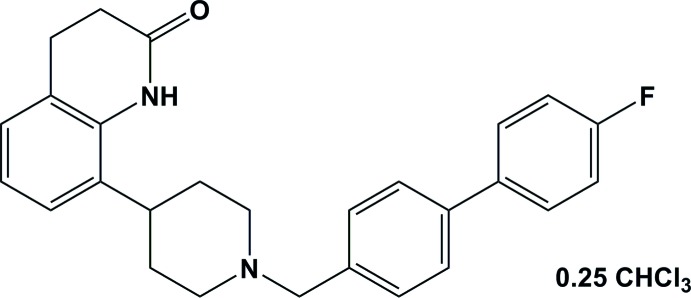


## Experimental   

### 

#### Crystal data  

C_27_H_27_FN_2_O·0.25CHCl_3_
*M* *_r_* = 444.35
Triclinic, 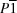

*a* = 7.6955 (8) Å
*b* = 16.618 (2) Å
*c* = 18.224 (2) Å
α = 79.206 (9)°
β = 87.563 (9)°
γ = 83.976 (9)°
*V* = 2276.1 (4) Å^3^
*Z* = 4
Mo *K*α radiation
μ = 0.17 mm^−1^
*T* = 173 K
0.45 × 0.30 × 0.15 mm

#### Data collection  

Stoe IPDS 2 diffractometer
Absorption correction: multi-scan (*MULscanABS* in *PLATON*; Spek, 2009 ▶) *T*_min_ = 0.764, *T*_max_ = 1.000
27043 measured reflections
8614 independent reflections
3481 reflections with *I* > 2σ(*I*)
*R*_int_ = 0.093

#### Refinement  

*R*[*F* ^2^ > 2σ(*F* ^2^)] = 0.050
*wR*(*F* ^2^) = 0.115
*S* = 0.69
8614 reflections
595 parameters
H atoms treated by a mixture of independent and constrained refinement
Δρ_max_ = 0.63 e Å^−3^
Δρ_min_ = −0.60 e Å^−3^

### 

Data collection: *X-AREA* (Stoe & Cie, 2009 ▶); cell refinement: *X-AREA*; data reduction: *X-RED32* (Stoe & Cie, 2009 ▶); program(s) used to solve structure: *SHELXS97* (Sheldrick, 2008 ▶); program(s) used to refine structure: *SHELXL2013* (Sheldrick, 2008 ▶); molecular graphics: *Mercury* (Macrae *et al.*, 2008 ▶); software used to prepare material for publication: *SHELXL2013* (Sheldrick, 2008 ▶), *PLATON* (Spek, 2009 ▶) and *publCIF* (Westrip, 2010 ▶).

## Supplementary Material

Crystal structure: contains datablock(s) I, global. DOI: 10.1107/S160053681303448X/lr2120sup1.cif

Structure factors: contains datablock(s) I. DOI: 10.1107/S160053681303448X/lr2120Isup2.hkl

Supporting information file. DOI: 10.1107/S160053681303448X/lr2120Isup3.cml

CCDC reference:

Additional supporting information:  crystallographic information; 3D view; checkCIF report

## Figures and Tables

**Table 1:** Hydrogen-bond geometry (Å, °) *Cg*1, *Cg*2, *Cg*3, *Cg*4, *Cg*5 and *Cg*6 are the centroids of rings C2–C7, C8–C11/C27/C28, C16/C17/C21–C24, C29–C34, C35–C38/C54/C55 and C43/C44/C48–C51, respectively.

*D*—H⋯*A*	*D*—H	H⋯*A*	*D*⋯*A*	*D*—H⋯*A*
N2—H2*N*⋯O1^i^	0.88 (3)	1.99 (3)	2.869 (3)	173 (3)
N4—H4*N*⋯O2^ii^	0.85 (3)	2.01 (3)	2.854 (3)	169 (3)
C15—H15⋯O1^i^	1.00	2.42	3.286 (4)	145
C34—H34⋯F1^iii^	0.95	2.53	3.406 (4)	154
C42—H42⋯O2^ii^	1.00	2.34	3.233 (4)	148
C61—H61⋯N3^iv^	1.00	2.00	2.978 (9)	164
C3—H3⋯*Cg*3^v^	0.95	2.65	3.483 (3)	147
C7—H7⋯*Cg*4^iv^	0.95	2.82	3.366 (3)	117
C19—H19*B*⋯*Cg*2^i^	0.99	2.84	3.779 (3)	159
C26—H26*B*⋯*Cg*5^vi^	0.99	2.94	3.903 (3)	165
C28—H28⋯*Cg*5^iv^	0.95	2.71	3.567 (3)	150
C30—H30⋯*Cg*6^vii^	0.95	2.78	3.593 (4)	144
C46—H46*A*⋯*Cg*5^ii^	0.99	2.93	3.871 (3)	159
C53—H53*A*⋯*Cg*6^vi^	0.99	2.86	3.825 (3)	166
C55—H55⋯*Cg*1	0.95	2.72	3.580 (3)	151

Symmetry codes: (i) 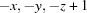; (ii) 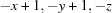; (iii) 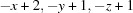; (iv) 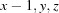; (v) 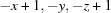; (vi) 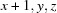; (vii) 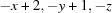.

**Fig. 1.**
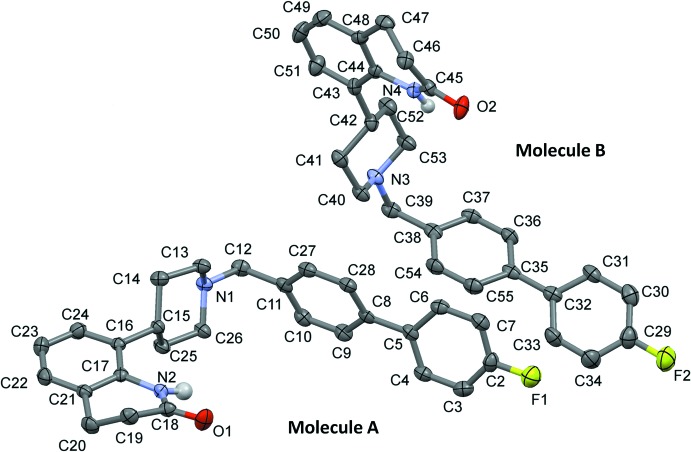
A view of the two independent molecules (A and B) of the title compound. Displacement ellipsoids are drawn at the 50% probability level. The C-bound H atoms and the disordered chloroform molecule of solvent has been omitted for clarity.

**Fig. 2.**
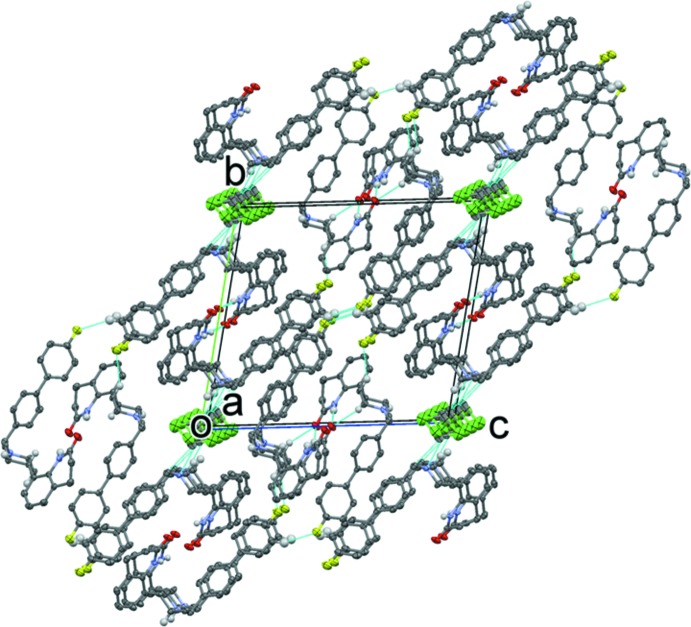
A partial view along the a axis of the crystal packing of the title compound. The hydrogen bonds are shown as dashed lines (see Table 1 for details; H atoms not involved in hydrogen bonding have been omitted for clarity).

## supplementary crystallographic information

### 1.1. Synthesis and crystallization

The synthesis of the title compound has been previously described (Ullah, 2012; Eastwood, 2000). Rod-like colourless crystals of the title compound were obtained by slow evaporation of a solution in chloro­form.

### 1.2. Refinement

Crystal data, data collection and structure refinement details are summarized in Table 1. The NH H atoms were located in a difference Fourier map and freely refined. The C-bound H atoms were included in calculated positions and treated as riding atoms: C—H = 0.95, 1.00 and 0.99 Å for CH(aromatic), methine, and methyl­ene H atoms, respectives, with *U*_iso_(H) = 1.2*U*_eq_(C).

### 2. Comment

In ongoing efforts to develop new anti­psychotics, we have synthesized a series of compounds which are structural analogs of adoprazine and bifeprunox and have disclosed their dual D2 and 5-HT_1A_ receptor binding affinities and structure-activity relationship (Ullah, 2013; Ullah & Al-Shaheri, 2012). Herein, we describe the crystal structure of one such molecule, a piperidine quinoline derivative.

The molecular structure of the two independent moleules (A and B) of the title compound are illustrated in Fig. 1. The compound crystallizes with a partially disordered chloro­form molecule situated about an inversion center. The bond lengths (Allen *et al.*, 1987) and bond angles are within normal values.

The conformation of the two molecules is very similar. The piperidine rings, N1/C13—C15/C25/C26 and N3/C40—C42/C52/C53, in molecules A and B, respectively, each have a chair conformation and their mean planes are inclined to the benzene ring to which they are attached by 80.06 (14) ° in molecule A and 83.75 (15)° in molecule B. The piperidin-2-one rings of the quinoline moiety, N2/C17—C21 in A and N4/C44—C48 in B, have screw boat conformations. The two benzene rings of the bi­phenyl moiety are inclined to one another by 26.37 (14) and 23.75 (15) ° in molecules A and B, respectively.

In the crystal, individual molecules are linked by pairs of N—H···O hydrogen bonds forming A—A and B—B inversion dimers (Table 1 and Fig. 2), with *R*^2^_2_(8) ring motifs (Bernstein *et al.*, 1995). The dimers, are stabilized by C—H···O hydrogen bonds, and linked *via* C—H···F and C—H···N hydrogen bonds forming a three-dimensional network (Fig. 2). The network is further stabilized by a number of C—H···π inter­actions (Table 1).

### Crystal data

**Table:**

C_27_H_27_FN_2_O·0.25CHCl_3_	*Z* = 4
*M**_r_* = 444.35	*F*(000) = 938
Triclinic, *P*1	*D*_x_ = 1.297 Mg m^−^^3^
Hall symbol: -P 1	Mo *K*α radiation, λ = 0.71073 Å
*a* = 7.6955 (8) Å	Cell parameters from 8114 reflections
*b* = 16.618 (2) Å	θ = 1.5–26.0°
*c* = 18.224 (2) Å	µ = 0.17 mm^−^^1^
α = 79.206 (9)°	*T* = 173 K
β = 87.563 (9)°	Rod, colourless
γ = 83.976 (9)°	0.45 × 0.30 × 0.15 mm
*V* = 2276.1 (4) Å^3^	

### Data collection

**Table:**

Stoe IPDS 2 diffractometer	8614 independent reflections
Radiation source: fine-focus sealed tube	3481 reflections with *I* > 2σ(*I*)
Plane graphite monochromator	*R*_int_ = 0.093
φ + ω scans	θ_max_ = 25.7°, θ_min_ = 1.5°
Absorption correction: multi-scan (MULscanABS in *PLATON*; Spek, 2009)	*h* = −9→9
*T*_min_ = 0.764, *T*_max_ = 1.000	*k* = −20→20
27043 measured reflections	*l* = −22→22

### Refinement

**Table:**

Refinement on *F*^2^	Hydrogen site location: mixed
Least-squares matrix: full	H atoms treated by a mixture of independent and constrained refinement
*R*[*F*^2^ > 2σ(*F*^2^)] = 0.050	*w* = 1/[σ^2^(*F*_o_^2^) + (0.0494*P*)^2^] where *P* = (*F*_o_^2^ + 2*F*_c_^2^)/3
*wR*(*F*^2^) = 0.115	(Δ/σ)_max_ < 0.001
*S* = 0.69	Δρ_max_ = 0.63 e Å^−^^3^
8614 reflections	Δρ_min_ = −0.60 e Å^−^^3^
595 parameters	Extinction correction: *SHELXL2013* (Sheldrick, 2008), Fc^*^=kFc[1+0.001xFc^2^λ^3^/sin(2θ)]^-1/4^
0 restraints	Extinction coefficient: 0.0034 (5)

### Special details

**Table:**

Geometry. All e.s.d.'s (except the e.s.d. in the dihedral angle between two l.s. planes) are estimated using the full covariance matrix. The cell e.s.d.'s are taken into account individually in the estimation of e.s.d.'s in distances, angles and torsion angles; correlations between e.s.d.'s in cell parameters are only used when they are defined by crystal symmetry. An approximate (isotropic) treatment of cell e.s.d.'s is used for estimating e.s.d.'s involving l.s. planes.
Refinement. The crystal diffracted weakly beyond 19° in θ despite is size. This we believe is due to the presence of disordered solvent of crystallization.

### Fractional atomic coordinates and isotropic or equivalent isotropic displacement parameters (Å^2^)

**Table:**

	*x*	*y*	*z*	*U*_iso_*/*U*_eq_	Occ. (<1)
F1	0.7833 (3)	0.46774 (11)	0.43761 (10)	0.0520 (6)	
O1	−0.2054 (3)	0.00085 (13)	0.53754 (12)	0.0385 (6)	
N1	0.4532 (3)	−0.07591 (14)	0.25646 (12)	0.0246 (6)	
N2	−0.0871 (3)	−0.09665 (15)	0.47441 (13)	0.0232 (6)	
H2N	0.009 (4)	−0.0712 (18)	0.4712 (16)	0.034 (9)*	
C2	0.7566 (4)	0.39930 (19)	0.40904 (18)	0.0326 (8)	
C3	0.8092 (4)	0.32282 (19)	0.44942 (17)	0.0346 (8)	
H3	0.8624	0.3169	0.4965	0.042*	
C4	0.7826 (4)	0.25500 (19)	0.41952 (16)	0.0292 (8)	
H4	0.8186	0.2017	0.4468	0.035*	
C5	0.7046 (4)	0.26184 (17)	0.35058 (16)	0.0239 (7)	
C6	0.6540 (4)	0.34185 (18)	0.31198 (16)	0.0282 (7)	
H6	0.6013	0.3490	0.2647	0.034*	
C7	0.6794 (4)	0.41019 (19)	0.34164 (17)	0.0321 (8)	
H7	0.6436	0.4640	0.3154	0.039*	
C8	0.6745 (4)	0.18862 (17)	0.31910 (15)	0.0246 (7)	
C9	0.7798 (4)	0.11402 (18)	0.33780 (16)	0.0301 (8)	
H9	0.8726	0.1101	0.3715	0.036*	
C10	0.7510 (4)	0.04604 (18)	0.30805 (16)	0.0284 (7)	
H10	0.8233	−0.0039	0.3227	0.034*	
C11	0.6193 (4)	0.04847 (18)	0.25737 (16)	0.0256 (7)	
C12	0.5943 (4)	−0.02544 (18)	0.22348 (16)	0.0295 (8)	
H12A	0.5712	−0.0061	0.1697	0.035*	
H12B	0.7057	−0.0615	0.2271	0.035*	
C13	0.2788 (4)	−0.03846 (18)	0.23558 (16)	0.0286 (7)	
H13A	0.2746	−0.0207	0.1807	0.034*	
H13B	0.2503	0.0108	0.2588	0.034*	
C14	0.1447 (4)	−0.09995 (18)	0.26140 (15)	0.0284 (7)	
H14A	0.1728	−0.1493	0.2383	0.034*	
H14B	0.0266	−0.0748	0.2458	0.034*	
C15	0.1482 (4)	−0.12460 (17)	0.34640 (15)	0.0234 (7)	
H15	0.1244	−0.0730	0.3674	0.028*	
C16	0.0118 (4)	−0.18178 (17)	0.38047 (15)	0.0219 (7)	
C17	−0.0964 (4)	−0.16765 (17)	0.44165 (15)	0.0227 (7)	
C18	−0.2169 (4)	−0.06471 (19)	0.51550 (16)	0.0268 (7)	
C19	−0.3732 (4)	−0.11199 (18)	0.53351 (17)	0.0303 (8)	
H19A	−0.4592	−0.0943	0.4933	0.036*	
H19B	−0.4293	−0.1003	0.5808	0.036*	
C20	−0.3202 (4)	−0.20388 (18)	0.54119 (17)	0.0335 (8)	
H20A	−0.2500	−0.2233	0.5865	0.040*	
H20B	−0.4263	−0.2336	0.5470	0.040*	
C21	−0.2159 (4)	−0.22272 (18)	0.47433 (15)	0.0250 (7)	
C22	−0.2296 (4)	−0.29239 (19)	0.44420 (17)	0.0319 (8)	
H22	−0.3118	−0.3297	0.4651	0.038*	
C23	−0.1250 (4)	−0.30808 (19)	0.38413 (17)	0.0358 (8)	
H23	−0.1343	−0.3562	0.3640	0.043*	
C24	−0.0059 (4)	−0.25301 (18)	0.35321 (17)	0.0318 (8)	
H24	0.0658	−0.2645	0.3120	0.038*	
C25	0.3338 (4)	−0.16133 (18)	0.36797 (16)	0.0299 (8)	
H25A	0.3407	−0.1750	0.4231	0.036*	
H25B	0.3613	−0.2130	0.3483	0.036*	
C26	0.4679 (4)	−0.10162 (18)	0.33715 (15)	0.0280 (7)	
H26A	0.4492	−0.0527	0.3613	0.034*	
H26B	0.5870	−0.1285	0.3489	0.034*	
C27	0.5138 (4)	0.12268 (18)	0.23943 (16)	0.0293 (8)	
H27	0.4217	0.1265	0.2055	0.035*	
C28	0.5396 (4)	0.19087 (18)	0.26978 (15)	0.0264 (7)	
H28	0.4639	0.2401	0.2567	0.032*	
F2	1.3617 (3)	0.62544 (12)	0.38567 (11)	0.0588 (6)	
O2	0.2877 (3)	0.54027 (12)	−0.01363 (13)	0.0377 (6)	
N3	0.9771 (3)	0.20169 (14)	0.05422 (13)	0.0270 (6)	
N4	0.4096 (3)	0.42911 (14)	−0.05795 (14)	0.0229 (6)	
H4N	0.502 (4)	0.4311 (17)	−0.0344 (15)	0.022 (8)*	
C29	1.3188 (5)	0.5689 (2)	0.3462 (2)	0.0410 (9)	
C30	1.3440 (4)	0.5841 (2)	0.27003 (19)	0.0393 (9)	
H30	1.3864	0.6341	0.2452	0.047*	
C31	1.3064 (4)	0.52515 (18)	0.23002 (17)	0.0319 (8)	
H31	1.3235	0.5351	0.1773	0.038*	
C32	1.2437 (4)	0.45105 (18)	0.26552 (16)	0.0283 (7)	
C33	1.2175 (4)	0.44066 (19)	0.34284 (18)	0.0362 (8)	
H33	1.1721	0.3918	0.3684	0.043*	
C34	1.2546 (5)	0.4982 (2)	0.38355 (19)	0.0421 (9)	
H34	1.2362	0.4893	0.4362	0.051*	
C35	1.2088 (4)	0.38639 (18)	0.22319 (16)	0.0273 (7)	
C36	1.2923 (4)	0.37995 (18)	0.15466 (16)	0.0297 (8)	
H36	1.3708	0.4190	0.1337	0.036*	
C37	1.2634 (4)	0.31827 (18)	0.11671 (16)	0.0299 (8)	
H37	1.3212	0.3163	0.0699	0.036*	
C38	1.1510 (4)	0.25863 (18)	0.14560 (16)	0.0277 (7)	
C39	1.1252 (4)	0.18953 (18)	0.10479 (16)	0.0300 (8)	
H39A	1.2334	0.1787	0.0754	0.036*	
H39B	1.1117	0.1393	0.1427	0.036*	
C40	0.8066 (4)	0.20082 (18)	0.09254 (16)	0.0281 (7)	
H40A	0.7850	0.2494	0.1171	0.034*	
H40B	0.8058	0.1508	0.1317	0.034*	
C41	0.6633 (4)	0.20212 (18)	0.03805 (16)	0.0298 (7)	
H41A	0.5490	0.2004	0.0650	0.036*	
H41B	0.6834	0.1530	0.0141	0.036*	
C42	0.6609 (4)	0.28031 (17)	−0.02180 (16)	0.0256 (7)	
H42	0.6425	0.3284	0.0045	0.031*	
C43	0.5169 (4)	0.29027 (17)	−0.07819 (16)	0.0253 (7)	
C44	0.4001 (4)	0.36189 (17)	−0.09489 (15)	0.0221 (7)	
C45	0.2736 (4)	0.48745 (18)	−0.05182 (16)	0.0268 (7)	
C46	0.1135 (4)	0.48381 (18)	−0.09304 (16)	0.0298 (8)	
H46A	0.0512	0.5397	−0.1055	0.036*	
H46B	0.0348	0.4480	−0.0608	0.036*	
C47	0.1570 (4)	0.45047 (18)	−0.16440 (16)	0.0331 (8)	
H47A	0.0477	0.4413	−0.1870	0.040*	
H47B	0.2170	0.4913	−0.2006	0.040*	
C48	0.2733 (4)	0.37045 (18)	−0.14847 (15)	0.0244 (7)	
C49	0.2579 (4)	0.3066 (2)	−0.18601 (17)	0.0357 (8)	
H49	0.1688	0.3113	−0.2215	0.043*	
C50	0.3729 (5)	0.2356 (2)	−0.17158 (18)	0.0406 (9)	
H50	0.3650	0.1922	−0.1982	0.049*	
C51	0.4987 (4)	0.22842 (19)	−0.11845 (18)	0.0365 (8)	
H51	0.5758	0.1793	−0.1090	0.044*	
C52	0.8422 (4)	0.28231 (19)	−0.06015 (17)	0.0343 (8)	
H52A	0.8639	0.2359	−0.0874	0.041*	
H52B	0.8460	0.3342	−0.0969	0.041*	
C53	0.9835 (4)	0.27626 (19)	−0.00341 (16)	0.0323 (8)	
H53A	1.0994	0.2752	−0.0291	0.039*	
H53B	0.9680	0.3254	0.0205	0.039*	
C54	1.0668 (4)	0.26511 (19)	0.21371 (16)	0.0310 (8)	
H54	0.9889	0.2258	0.2348	0.037*	
C55	1.0941 (4)	0.32725 (19)	0.25114 (17)	0.0321 (8)	
H55	1.0333	0.3301	0.2972	0.039*	
Cl1	−0.0132 (2)	−0.03511 (9)	0.07915 (8)	0.1134 (6)	
Cl2	0.3096 (5)	0.02001 (16)	0.00809 (15)	0.1066 (10)	0.5
C61	0.0799 (10)	0.0456 (5)	−0.0014 (4)	0.065 (3)	0.5
H61	0.0458	0.1028	0.0074	0.078*	0.5

### Atomic displacement parameters (Å^2^)

**Table:**

	*U*^11^	*U*^22^	*U*^33^	*U*^12^	*U*^13^	*U*^23^
F1	0.0699 (16)	0.0417 (12)	0.0472 (12)	−0.0041 (11)	−0.0038 (11)	−0.0153 (9)
O1	0.0344 (15)	0.0437 (14)	0.0431 (13)	−0.0094 (11)	0.0146 (11)	−0.0231 (11)
N1	0.0206 (15)	0.0286 (14)	0.0239 (13)	−0.0027 (11)	0.0034 (11)	−0.0039 (11)
N2	0.0214 (16)	0.0278 (14)	0.0214 (13)	−0.0080 (12)	0.0059 (12)	−0.0053 (11)
C2	0.034 (2)	0.0292 (18)	0.0378 (19)	−0.0041 (15)	0.0051 (16)	−0.0143 (15)
C3	0.036 (2)	0.042 (2)	0.0248 (17)	−0.0015 (17)	−0.0018 (15)	−0.0050 (15)
C4	0.028 (2)	0.0304 (18)	0.0256 (17)	0.0000 (15)	0.0004 (15)	0.0024 (14)
C5	0.0143 (17)	0.0274 (17)	0.0272 (16)	−0.0016 (13)	0.0046 (13)	0.0008 (13)
C6	0.0225 (19)	0.0342 (18)	0.0252 (16)	0.0005 (14)	−0.0013 (14)	−0.0007 (14)
C7	0.031 (2)	0.0285 (18)	0.0326 (18)	0.0009 (15)	0.0026 (16)	0.0016 (14)
C8	0.0224 (18)	0.0273 (17)	0.0208 (16)	−0.0038 (14)	0.0057 (14)	0.0037 (13)
C9	0.0227 (19)	0.0321 (18)	0.0326 (18)	−0.0013 (15)	−0.0010 (15)	0.0007 (14)
C10	0.0239 (19)	0.0255 (17)	0.0325 (18)	0.0013 (14)	0.0040 (15)	0.0004 (14)
C11	0.0199 (18)	0.0294 (17)	0.0252 (16)	−0.0046 (14)	0.0067 (14)	0.0008 (13)
C12	0.0224 (19)	0.0355 (18)	0.0288 (17)	0.0000 (15)	0.0056 (14)	−0.0047 (14)
C13	0.0276 (19)	0.0342 (18)	0.0224 (16)	−0.0037 (15)	0.0004 (14)	−0.0008 (13)
C14	0.0255 (19)	0.0346 (18)	0.0241 (16)	−0.0009 (15)	−0.0008 (14)	−0.0038 (13)
C15	0.0262 (19)	0.0217 (16)	0.0221 (15)	−0.0038 (14)	0.0021 (14)	−0.0036 (12)
C16	0.0207 (18)	0.0253 (16)	0.0191 (15)	−0.0026 (13)	−0.0024 (13)	−0.0022 (13)
C17	0.0220 (18)	0.0223 (16)	0.0234 (16)	−0.0012 (13)	−0.0049 (14)	−0.0028 (13)
C18	0.0237 (19)	0.0329 (18)	0.0237 (16)	−0.0031 (15)	0.0029 (14)	−0.0051 (14)
C19	0.0236 (19)	0.0388 (19)	0.0290 (17)	−0.0057 (15)	0.0076 (14)	−0.0076 (14)
C20	0.031 (2)	0.0373 (19)	0.0302 (18)	−0.0106 (16)	0.0086 (15)	−0.0002 (14)
C21	0.0227 (19)	0.0294 (17)	0.0213 (16)	−0.0057 (14)	−0.0007 (14)	0.0011 (13)
C22	0.030 (2)	0.0321 (19)	0.0339 (18)	−0.0129 (15)	−0.0029 (16)	−0.0012 (15)
C23	0.043 (2)	0.0285 (18)	0.039 (2)	−0.0099 (16)	−0.0018 (17)	−0.0094 (15)
C24	0.034 (2)	0.0345 (19)	0.0287 (17)	−0.0047 (16)	−0.0002 (15)	−0.0088 (15)
C25	0.030 (2)	0.0294 (17)	0.0280 (17)	−0.0042 (15)	−0.0040 (15)	0.0027 (13)
C26	0.0262 (19)	0.0284 (17)	0.0261 (16)	−0.0036 (14)	−0.0007 (14)	0.0041 (13)
C27	0.025 (2)	0.0354 (19)	0.0243 (16)	−0.0023 (15)	−0.0008 (14)	0.0028 (14)
C28	0.0244 (19)	0.0280 (17)	0.0234 (16)	0.0000 (14)	0.0005 (14)	0.0023 (13)
F2	0.0776 (17)	0.0417 (12)	0.0624 (14)	−0.0126 (11)	0.0063 (12)	−0.0215 (11)
O2	0.0299 (14)	0.0277 (12)	0.0601 (15)	0.0020 (10)	−0.0085 (12)	−0.0207 (12)
N3	0.0241 (15)	0.0250 (14)	0.0285 (14)	0.0009 (12)	0.0010 (12)	0.0011 (11)
N4	0.0198 (16)	0.0209 (14)	0.0279 (14)	−0.0004 (12)	−0.0049 (12)	−0.0042 (11)
C29	0.043 (2)	0.033 (2)	0.050 (2)	−0.0023 (17)	0.0032 (18)	−0.0158 (17)
C30	0.034 (2)	0.0284 (18)	0.052 (2)	0.0012 (16)	0.0015 (18)	−0.0027 (17)
C31	0.028 (2)	0.0289 (18)	0.0334 (18)	0.0002 (15)	0.0020 (15)	0.0059 (15)
C32	0.0228 (19)	0.0303 (17)	0.0277 (17)	0.0029 (14)	0.0024 (14)	0.0009 (14)
C33	0.038 (2)	0.0308 (18)	0.0366 (19)	−0.0049 (16)	0.0088 (16)	0.0011 (15)
C34	0.053 (3)	0.037 (2)	0.037 (2)	−0.0014 (18)	0.0091 (18)	−0.0112 (17)
C35	0.0188 (18)	0.0306 (18)	0.0267 (17)	0.0031 (14)	0.0007 (14)	0.0062 (14)
C36	0.026 (2)	0.0289 (17)	0.0308 (18)	−0.0031 (15)	0.0048 (15)	0.0016 (14)
C37	0.0256 (19)	0.0353 (19)	0.0242 (16)	0.0025 (15)	0.0035 (14)	0.0024 (14)
C38	0.0176 (18)	0.0297 (18)	0.0310 (18)	0.0027 (14)	−0.0036 (14)	0.0048 (14)
C39	0.0243 (19)	0.0326 (18)	0.0297 (17)	0.0035 (15)	−0.0003 (15)	−0.0005 (14)
C40	0.0236 (19)	0.0277 (17)	0.0290 (17)	−0.0023 (14)	0.0038 (14)	0.0038 (13)
C41	0.0272 (19)	0.0262 (17)	0.0335 (17)	−0.0010 (14)	0.0046 (15)	−0.0007 (13)
C42	0.0259 (19)	0.0174 (15)	0.0327 (17)	0.0014 (14)	−0.0017 (15)	−0.0045 (13)
C43	0.0255 (19)	0.0211 (16)	0.0300 (17)	−0.0033 (14)	0.0008 (14)	−0.0061 (13)
C44	0.0225 (18)	0.0248 (16)	0.0197 (15)	−0.0058 (14)	0.0053 (13)	−0.0050 (12)
C45	0.027 (2)	0.0199 (16)	0.0312 (17)	−0.0012 (14)	0.0022 (15)	−0.0016 (14)
C46	0.0243 (19)	0.0268 (17)	0.0376 (18)	0.0020 (14)	−0.0048 (15)	−0.0064 (14)
C47	0.031 (2)	0.0364 (19)	0.0283 (17)	−0.0003 (15)	−0.0059 (15)	0.0025 (14)
C48	0.0232 (19)	0.0299 (17)	0.0195 (15)	−0.0026 (14)	0.0025 (14)	−0.0038 (13)
C49	0.034 (2)	0.045 (2)	0.0306 (18)	−0.0065 (17)	−0.0046 (16)	−0.0130 (16)
C50	0.045 (2)	0.038 (2)	0.045 (2)	−0.0045 (18)	−0.0018 (18)	−0.0236 (17)
C51	0.036 (2)	0.0311 (19)	0.045 (2)	0.0018 (16)	−0.0028 (17)	−0.0153 (16)
C52	0.029 (2)	0.0350 (19)	0.0353 (19)	−0.0037 (15)	−0.0014 (16)	0.0027 (14)
C53	0.0258 (19)	0.0354 (18)	0.0305 (18)	−0.0044 (15)	−0.0002 (15)	0.0080 (14)
C54	0.025 (2)	0.0368 (19)	0.0284 (17)	−0.0086 (15)	0.0061 (15)	0.0021 (15)
C55	0.026 (2)	0.040 (2)	0.0279 (17)	−0.0042 (16)	0.0051 (15)	−0.0010 (15)
Cl1	0.1568 (15)	0.1040 (11)	0.0755 (9)	−0.0122 (10)	−0.0064 (9)	−0.0063 (8)
Cl2	0.157 (3)	0.0827 (18)	0.0861 (18)	0.0011 (19)	0.0029 (19)	−0.0392 (14)
C61	0.055 (6)	0.074 (6)	0.065 (5)	0.038 (5)	−0.047 (5)	−0.027 (4)

### Geometric parameters (Å, º)

**Table:**

F1—C2	1.374 (3)	N3—C39	1.470 (4)
O1—C18	1.242 (3)	N3—C53	1.470 (3)
N1—C13	1.454 (4)	N4—C45	1.366 (4)
N1—C26	1.458 (3)	N4—C44	1.417 (4)
N1—C12	1.480 (4)	N4—H4N	0.86 (3)
N2—C18	1.347 (4)	C29—C30	1.372 (5)
N2—C17	1.428 (4)	C29—C34	1.372 (4)
N2—H2N	0.88 (3)	C30—C31	1.386 (4)
C2—C7	1.360 (4)	C30—H30	0.9500
C2—C3	1.374 (4)	C31—C32	1.403 (4)
C3—C4	1.377 (4)	C31—H31	0.9500
C3—H3	0.9500	C32—C33	1.396 (4)
C4—C5	1.396 (4)	C32—C35	1.487 (4)
C4—H4	0.9500	C33—C34	1.374 (4)
C5—C6	1.407 (4)	C33—H33	0.9500
C5—C8	1.482 (4)	C34—H34	0.9500
C6—C7	1.381 (4)	C35—C36	1.397 (4)
C6—H6	0.9500	C35—C55	1.401 (4)
C7—H7	0.9500	C36—C37	1.380 (4)
C8—C28	1.395 (4)	C36—H36	0.9500
C8—C9	1.402 (4)	C37—C38	1.398 (4)
C9—C10	1.383 (4)	C37—H37	0.9500
C9—H9	0.9500	C38—C54	1.392 (4)
C10—C11	1.392 (4)	C38—C39	1.514 (4)
C10—H10	0.9500	C39—H39A	0.9900
C11—C27	1.396 (4)	C39—H39B	0.9900
C11—C12	1.507 (4)	C40—C41	1.511 (4)
C12—H12A	0.9900	C40—H40A	0.9900
C12—H12B	0.9900	C40—H40B	0.9900
C13—C14	1.526 (4)	C41—C42	1.531 (4)
C13—H13A	0.9900	C41—H41A	0.9900
C13—H13B	0.9900	C41—H41B	0.9900
C14—C15	1.527 (4)	C42—C43	1.519 (4)
C14—H14A	0.9900	C42—C52	1.534 (4)
C14—H14B	0.9900	C42—H42	1.0000
C15—C16	1.524 (4)	C43—C51	1.391 (4)
C15—C25	1.531 (4)	C43—C44	1.407 (4)
C15—H15	1.0000	C44—C48	1.388 (4)
C16—C24	1.388 (4)	C45—C46	1.482 (4)
C16—C17	1.403 (4)	C46—C47	1.518 (4)
C17—C21	1.401 (4)	C46—H46A	0.9900
C18—C19	1.497 (4)	C46—H46B	0.9900
C19—C20	1.520 (4)	C47—C48	1.510 (4)
C19—H19A	0.9900	C47—H47A	0.9900
C19—H19B	0.9900	C47—H47B	0.9900
C20—C21	1.495 (4)	C48—C49	1.383 (4)
C20—H20A	0.9900	C49—C50	1.389 (4)
C20—H20B	0.9900	C49—H49	0.9500
C21—C22	1.386 (4)	C50—C51	1.378 (4)
C22—C23	1.379 (4)	C50—H50	0.9500
C22—H22	0.9500	C51—H51	0.9500
C23—C24	1.391 (4)	C52—C53	1.515 (4)
C23—H23	0.9500	C52—H52A	0.9900
C24—H24	0.9500	C52—H52B	0.9900
C25—C26	1.524 (4)	C53—H53A	0.9900
C25—H25A	0.9900	C53—H53B	0.9900
C25—H25B	0.9900	C54—C55	1.377 (4)
C26—H26A	0.9900	C54—H54	0.9500
C26—H26B	0.9900	C55—H55	0.9500
C27—C28	1.386 (4)	Cl1—C61^i^	1.573 (7)
C27—H27	0.9500	Cl1—C61	1.960 (9)
C28—H28	0.9500	Cl2—C61	1.782 (8)
F2—C29	1.359 (4)	C61—Cl1^i^	1.573 (7)
O2—C45	1.234 (3)	C61—H61	1.0000
N3—C40	1.460 (4)		
			
C13—N1—C26	112.0 (2)	C45—N4—H4N	117.3 (19)
C13—N1—C12	113.8 (2)	C44—N4—H4N	118.5 (19)
C26—N1—C12	112.2 (2)	F2—C29—C30	118.6 (3)
C18—N2—C17	124.3 (3)	F2—C29—C34	119.2 (3)
C18—N2—H2N	114 (2)	C30—C29—C34	122.2 (3)
C17—N2—H2N	122 (2)	C29—C30—C31	118.7 (3)
C7—C2—F1	118.5 (3)	C29—C30—H30	120.7
C7—C2—C3	122.7 (3)	C31—C30—H30	120.7
F1—C2—C3	118.8 (3)	C30—C31—C32	121.6 (3)
C2—C3—C4	117.9 (3)	C30—C31—H31	119.2
C2—C3—H3	121.0	C32—C31—H31	119.2
C4—C3—H3	121.0	C33—C32—C31	116.6 (3)
C3—C4—C5	122.3 (3)	C33—C32—C35	121.6 (3)
C3—C4—H4	118.9	C31—C32—C35	121.8 (3)
C5—C4—H4	118.9	C34—C33—C32	122.7 (3)
C4—C5—C6	117.0 (3)	C34—C33—H33	118.7
C4—C5—C8	122.0 (3)	C32—C33—H33	118.7
C6—C5—C8	121.0 (3)	C29—C34—C33	118.3 (3)
C7—C6—C5	121.1 (3)	C29—C34—H34	120.9
C7—C6—H6	119.4	C33—C34—H34	120.9
C5—C6—H6	119.4	C36—C35—C55	116.5 (3)
C2—C7—C6	118.9 (3)	C36—C35—C32	121.9 (3)
C2—C7—H7	120.5	C55—C35—C32	121.6 (3)
C6—C7—H7	120.5	C37—C36—C35	121.6 (3)
C28—C8—C9	117.1 (3)	C37—C36—H36	119.2
C28—C8—C5	121.4 (3)	C35—C36—H36	119.2
C9—C8—C5	121.5 (3)	C36—C37—C38	121.5 (3)
C10—C9—C8	121.1 (3)	C36—C37—H37	119.3
C10—C9—H9	119.5	C38—C37—H37	119.3
C8—C9—H9	119.5	C54—C38—C37	117.0 (3)
C9—C10—C11	122.1 (3)	C54—C38—C39	121.9 (3)
C9—C10—H10	119.0	C37—C38—C39	121.0 (3)
C11—C10—H10	119.0	N3—C39—C38	117.5 (2)
C10—C11—C27	116.6 (3)	N3—C39—H39A	107.9
C10—C11—C12	121.2 (3)	C38—C39—H39A	107.9
C27—C11—C12	122.2 (3)	N3—C39—H39B	107.9
N1—C12—C11	116.8 (2)	C38—C39—H39B	107.9
N1—C12—H12A	108.1	H39A—C39—H39B	107.2
C11—C12—H12A	108.1	N3—C40—C41	110.7 (2)
N1—C12—H12B	108.1	N3—C40—H40A	109.5
C11—C12—H12B	108.1	C41—C40—H40A	109.5
H12A—C12—H12B	107.3	N3—C40—H40B	109.5
N1—C13—C14	110.1 (2)	C41—C40—H40B	109.5
N1—C13—H13A	109.6	H40A—C40—H40B	108.1
C14—C13—H13A	109.6	C40—C41—C42	109.9 (2)
N1—C13—H13B	109.6	C40—C41—H41A	109.7
C14—C13—H13B	109.6	C42—C41—H41A	109.7
H13A—C13—H13B	108.1	C40—C41—H41B	109.7
C13—C14—C15	109.1 (2)	C42—C41—H41B	109.7
C13—C14—H14A	109.9	H41A—C41—H41B	108.2
C15—C14—H14A	109.9	C43—C42—C41	114.7 (2)
C13—C14—H14B	109.9	C43—C42—C52	111.6 (2)
C15—C14—H14B	109.9	C41—C42—C52	108.1 (2)
H14A—C14—H14B	108.3	C43—C42—H42	107.4
C16—C15—C14	114.7 (2)	C41—C42—H42	107.4
C16—C15—C25	111.9 (2)	C52—C42—H42	107.4
C14—C15—C25	108.0 (2)	C51—C43—C44	116.4 (3)
C16—C15—H15	107.3	C51—C43—C42	120.5 (3)
C14—C15—H15	107.3	C44—C43—C42	123.1 (3)
C25—C15—H15	107.3	C48—C44—C43	121.9 (3)
C24—C16—C17	116.7 (3)	C48—C44—N4	117.2 (3)
C24—C16—C15	120.5 (3)	C43—C44—N4	120.8 (3)
C17—C16—C15	122.8 (3)	O2—C45—N4	120.0 (3)
C21—C17—C16	122.0 (3)	O2—C45—C46	123.0 (3)
C21—C17—N2	116.8 (3)	N4—C45—C46	117.0 (3)
C16—C17—N2	121.1 (3)	C45—C46—C47	111.3 (3)
O1—C18—N2	120.9 (3)	C45—C46—H46A	109.4
O1—C18—C19	122.0 (3)	C47—C46—H46A	109.4
N2—C18—C19	117.0 (3)	C45—C46—H46B	109.4
C18—C19—C20	110.5 (3)	C47—C46—H46B	109.4
C18—C19—H19A	109.5	H46A—C46—H46B	108.0
C20—C19—H19A	109.5	C48—C47—C46	110.7 (2)
C18—C19—H19B	109.5	C48—C47—H47A	109.5
C20—C19—H19B	109.5	C46—C47—H47A	109.5
H19A—C19—H19B	108.1	C48—C47—H47B	109.5
C21—C20—C19	111.1 (2)	C46—C47—H47B	109.5
C21—C20—H20A	109.4	H47A—C47—H47B	108.1
C19—C20—H20A	109.4	C49—C48—C44	119.6 (3)
C21—C20—H20B	109.4	C49—C48—C47	122.0 (3)
C19—C20—H20B	109.4	C44—C48—C47	118.3 (3)
H20A—C20—H20B	108.0	C48—C49—C50	119.8 (3)
C22—C21—C17	118.8 (3)	C48—C49—H49	120.1
C22—C21—C20	123.2 (3)	C50—C49—H49	120.1
C17—C21—C20	118.0 (3)	C51—C50—C49	119.8 (3)
C23—C22—C21	120.6 (3)	C51—C50—H50	120.1
C23—C22—H22	119.7	C49—C50—H50	120.1
C21—C22—H22	119.7	C50—C51—C43	122.5 (3)
C22—C23—C24	119.6 (3)	C50—C51—H51	118.7
C22—C23—H23	120.2	C43—C51—H51	118.7
C24—C23—H23	120.2	C53—C52—C42	110.9 (2)
C16—C24—C23	122.3 (3)	C53—C52—H52A	109.5
C16—C24—H24	118.8	C42—C52—H52A	109.5
C23—C24—H24	118.8	C53—C52—H52B	109.5
C26—C25—C15	111.4 (2)	C42—C52—H52B	109.5
C26—C25—H25A	109.3	H52A—C52—H52B	108.1
C15—C25—H25A	109.3	N3—C53—C52	111.1 (3)
C26—C25—H25B	109.3	N3—C53—H53A	109.4
C15—C25—H25B	109.3	C52—C53—H53A	109.4
H25A—C25—H25B	108.0	N3—C53—H53B	109.4
N1—C26—C25	110.8 (2)	C52—C53—H53B	109.4
N1—C26—H26A	109.5	H53A—C53—H53B	108.0
C25—C26—H26A	109.5	C55—C54—C38	121.4 (3)
N1—C26—H26B	109.5	C55—C54—H54	119.3
C25—C26—H26B	109.5	C38—C54—H54	119.3
H26A—C26—H26B	108.1	C54—C55—C35	121.9 (3)
C28—C27—C11	121.9 (3)	C54—C55—H55	119.1
C28—C27—H27	119.1	C35—C55—H55	119.1
C11—C27—H27	119.1	C61^i^—Cl1—C61	69.6 (5)
C27—C28—C8	121.2 (3)	Cl1^i^—C61—Cl2	112.9 (5)
C27—C28—H28	119.4	Cl1^i^—C61—Cl1	110.4 (5)
C8—C28—H28	119.4	Cl2—C61—Cl1	101.8 (3)
C40—N3—C39	113.9 (2)	Cl1^i^—C61—H61	110.5
C40—N3—C53	110.8 (2)	Cl2—C61—H61	110.5
C39—N3—C53	112.0 (2)	Cl1—C61—H61	110.5
C45—N4—C44	124.0 (3)		
			
C7—C2—C3—C4	0.3 (5)	F2—C29—C30—C31	177.6 (3)
F1—C2—C3—C4	−179.4 (3)	C34—C29—C30—C31	−1.3 (5)
C2—C3—C4—C5	−0.1 (5)	C29—C30—C31—C32	0.0 (5)
C3—C4—C5—C6	0.2 (5)	C30—C31—C32—C33	1.4 (5)
C3—C4—C5—C8	−179.3 (3)	C30—C31—C32—C35	−177.9 (3)
C4—C5—C6—C7	−0.5 (4)	C31—C32—C33—C34	−1.7 (5)
C8—C5—C6—C7	179.0 (3)	C35—C32—C33—C34	177.7 (3)
F1—C2—C7—C6	179.2 (3)	F2—C29—C34—C33	−177.9 (3)
C3—C2—C7—C6	−0.6 (5)	C30—C29—C34—C33	1.1 (5)
C5—C6—C7—C2	0.7 (5)	C32—C33—C34—C29	0.5 (5)
C4—C5—C8—C28	152.9 (3)	C33—C32—C35—C36	−155.2 (3)
C6—C5—C8—C28	−26.6 (4)	C31—C32—C35—C36	24.1 (4)
C4—C5—C8—C9	−26.8 (4)	C33—C32—C35—C55	23.0 (4)
C6—C5—C8—C9	153.7 (3)	C31—C32—C35—C55	−157.7 (3)
C28—C8—C9—C10	0.4 (4)	C55—C35—C36—C37	−0.2 (4)
C5—C8—C9—C10	−179.9 (3)	C32—C35—C36—C37	178.0 (3)
C8—C9—C10—C11	1.3 (5)	C35—C36—C37—C38	−0.9 (5)
C9—C10—C11—C27	−1.7 (4)	C36—C37—C38—C54	1.2 (4)
C9—C10—C11—C12	177.7 (3)	C36—C37—C38—C39	−177.8 (3)
C13—N1—C12—C11	75.3 (3)	C40—N3—C39—C38	−70.1 (3)
C26—N1—C12—C11	−53.1 (3)	C53—N3—C39—C38	56.6 (3)
C10—C11—C12—N1	100.2 (3)	C54—C38—C39—N3	86.9 (4)
C27—C11—C12—N1	−80.3 (4)	C37—C38—C39—N3	−94.1 (3)
C26—N1—C13—C14	−60.6 (3)	C39—N3—C40—C41	−172.4 (2)
C12—N1—C13—C14	170.9 (2)	C53—N3—C40—C41	60.3 (3)
N1—C13—C14—C15	61.2 (3)	N3—C40—C41—C42	−60.4 (3)
C13—C14—C15—C16	176.2 (2)	C40—C41—C42—C43	−177.6 (3)
C13—C14—C15—C25	−58.2 (3)	C40—C41—C42—C52	57.1 (3)
C14—C15—C16—C24	50.7 (4)	C41—C42—C43—C51	−54.7 (4)
C25—C15—C16—C24	−72.8 (3)	C52—C42—C43—C51	68.7 (4)
C14—C15—C16—C17	−131.7 (3)	C41—C42—C43—C44	127.7 (3)
C25—C15—C16—C17	104.8 (3)	C52—C42—C43—C44	−108.9 (3)
C24—C16—C17—C21	0.5 (4)	C51—C43—C44—C48	0.1 (4)
C15—C16—C17—C21	−177.2 (3)	C42—C43—C44—C48	177.8 (3)
C24—C16—C17—N2	179.3 (3)	C51—C43—C44—N4	−178.9 (3)
C15—C16—C17—N2	1.6 (4)	C42—C43—C44—N4	−1.2 (4)
C18—N2—C17—C21	−23.1 (4)	C45—N4—C44—C48	23.1 (4)
C18—N2—C17—C16	158.0 (3)	C45—N4—C44—C43	−157.8 (3)
C17—N2—C18—O1	−174.4 (3)	C44—N4—C45—O2	174.1 (3)
C17—N2—C18—C19	6.1 (4)	C44—N4—C45—C46	−6.3 (4)
O1—C18—C19—C20	−147.6 (3)	O2—C45—C46—C47	148.1 (3)
N2—C18—C19—C20	31.9 (4)	N4—C45—C46—C47	−31.5 (4)
C18—C19—C20—C21	−52.3 (3)	C45—C46—C47—C48	51.3 (3)
C16—C17—C21—C22	−1.3 (4)	C43—C44—C48—C49	1.2 (4)
N2—C17—C21—C22	179.8 (3)	N4—C44—C48—C49	−179.7 (3)
C16—C17—C21—C20	177.6 (3)	C43—C44—C48—C47	−178.2 (3)
N2—C17—C21—C20	−1.3 (4)	N4—C44—C48—C47	0.9 (4)
C19—C20—C21—C22	−143.2 (3)	C46—C47—C48—C49	143.6 (3)
C19—C20—C21—C17	38.0 (4)	C46—C47—C48—C44	−37.0 (4)
C17—C21—C22—C23	1.3 (4)	C44—C48—C49—C50	−2.1 (5)
C20—C21—C22—C23	−177.5 (3)	C47—C48—C49—C50	177.2 (3)
C21—C22—C23—C24	−0.6 (5)	C48—C49—C50—C51	1.8 (5)
C17—C16—C24—C23	0.3 (4)	C49—C50—C51—C43	−0.5 (5)
C15—C16—C24—C23	178.0 (3)	C44—C43—C51—C50	−0.5 (5)
C22—C23—C24—C16	−0.3 (5)	C42—C43—C51—C50	−178.3 (3)
C16—C15—C25—C26	−176.8 (3)	C43—C42—C52—C53	177.4 (2)
C14—C15—C25—C26	55.9 (3)	C41—C42—C52—C53	−55.5 (3)
C13—N1—C26—C25	57.2 (3)	C40—N3—C53—C52	−58.3 (3)
C12—N1—C26—C25	−173.5 (2)	C39—N3—C53—C52	173.4 (3)
C15—C25—C26—N1	−55.2 (3)	C42—C52—C53—N3	56.6 (3)
C10—C11—C27—C28	0.7 (4)	C37—C38—C54—C55	−0.4 (4)
C12—C11—C27—C28	−178.8 (3)	C39—C38—C54—C55	178.6 (3)
C11—C27—C28—C8	0.9 (5)	C38—C54—C55—C35	−0.7 (5)
C9—C8—C28—C27	−1.4 (4)	C36—C35—C55—C54	1.0 (4)
C5—C8—C28—C27	178.8 (3)	C32—C35—C55—C54	−177.2 (3)

Symmetry code: (i) −*x*, −*y*, −*z*.

### Hydrogen-bond geometry (Å, º)

Cg1, Cg2, Cg3, Cg4, Cg5 and Cg6 are the centroids of rings C2–C7, C8–C11/C27/C28, C16/C17/C21–C24, C29–C34, C35–C38/C54/C55 and C43/C44/C48–C51, respectively.

**Table:**

*D*—H···*A*	*D*—H	H···*A*	*D*···*A*	*D*—H···*A*
N2—H2*N*···O1^ii^	0.88 (3)	1.99 (3)	2.869 (3)	173 (3)
N4—H4*N*···O2^iii^	0.85 (3)	2.01 (3)	2.854 (3)	169 (3)
C15—H15···O1^ii^	1.00	2.42	3.286 (4)	145
C34—H34···F1^iv^	0.95	2.53	3.406 (4)	154
C42—H42···O2^iii^	1.00	2.34	3.233 (4)	148
C61—H61···N3^v^	1.00	2.00	2.978 (9)	164
C3—H3···*Cg*3^vi^	0.95	2.65	3.483 (3)	147
C7—H7···*Cg*4^v^	0.95	2.82	3.366 (3)	117
C19—H19*B*···*Cg*2^ii^	0.99	2.84	3.779 (3)	159
C26—H26*B*···*Cg*5^vii^	0.99	2.94	3.903 (3)	165
C28—H28···*Cg*5^v^	0.95	2.71	3.567 (3)	150
C30—H30···*Cg*6^viii^	0.95	2.78	3.593 (4)	144
C46—H46*A*···*Cg*5^iii^	0.99	2.93	3.871 (3)	159
C53—H53*A*···*Cg*6^vii^	0.99	2.86	3.825 (3)	166
C55—H55···*Cg*1	0.95	2.72	3.580 (3)	151

Symmetry codes: (ii) −*x*, −*y*, −*z*+1; (iii) −*x*+1, −*y*+1, −*z*; (iv) −*x*+2, −*y*+1, −*z*+1; (v) *x*−1, *y*, *z*; (vi) −*x*+1, −*y*, −*z*+1; (vii) *x*+1, *y*, *z*; (viii) −*x*+2, −*y*+1, −*z*.
